# Death of Sword Swallower

**Published:** 1917-02

**Authors:** 


**Death of Sword Swallower.** Weinert, Munch. Med. Woch., reports the case of a soldier who resumed his practice after the healing of a bullet wound of the thorax, fatal haemoptysis resulting. It appeared that in the process of cicatrization, the oesophagus was displaced so that the sword penetarted the aorta, at a point wheer resistance was encountered.

